# ICAM-1-related long non-coding RNA: promoter analysis and expression in human retinal endothelial cells

**DOI:** 10.1186/s13104-018-3384-8

**Published:** 2018-05-09

**Authors:** Amanda L. Lumsden, Yuefang Ma, Liam M. Ashander, Andrew J. Stempel, Damien J. Keating, Justine R. Smith, Binoy Appukuttan

**Affiliations:** Flinders University College of Medicine and Public Health, Flinders Medical Centre Room 4E-431, Flinders Drive, Bedford Park, SA 5042 Australia

**Keywords:** Intercellular adhesion molecule, ICAM-1, ICAM-1-related, Promoter

## Abstract

**Objective:**

Regulation of intercellular adhesion molecule (ICAM)-1 in retinal endothelial cells is a promising druggable target for retinal vascular diseases. The ICAM-1-related (ICR) long non-coding RNA stabilizes ICAM-1 transcript, increasing protein expression. However, studies of ICR involvement in disease have been limited as the promoter is uncharacterized. To address this issue, we undertook a comprehensive in silico analysis of the human *ICR* gene promoter region.

**Results:**

We used genomic evolutionary rate profiling to identify a 115 base pair (bp) sequence within 500 bp upstream of the transcription start site of the annotated human *ICR* gene that was conserved across 25 eutherian genomes. A second constrained sequence upstream of the orthologous mouse gene (68 bp; conserved across 27 Eutherian genomes including human) was also discovered. Searching these elements identified 33 matrices predictive of binding sites for transcription factors known to be responsive to a broad range of pathological stimuli, including hypoxia, and metabolic and inflammatory proteins. Five phenotype-associated single nucleotide polymorphisms (SNPs) in the immediate vicinity of these elements included four SNPs (i.e. rs2569693, rs281439, rs281440 and rs11575074) predicted to impact binding motifs of transcription factors, and thus the expression of *ICR* and *ICAM*-*1* genes, with potential to influence disease susceptibility. We verified that human retinal endothelial cells expressed ICR, and observed induction of expression by tumor necrosis factor-α.

**Electronic supplementary material:**

The online version of this article (10.1186/s13104-018-3384-8) contains supplementary material, which is available to authorized users.

## Introduction

### Background

Retinal vascular diseases occur commonly and represent a major cause of blindness in developing and developed nations. These conditions include diabetic retinopathy, major retinal vessel occlusions, sickle cell retinopathy, retinopathy of prematurity and uveitis [1]. A key feature of the pathology of any retinal vasculopathy is over-expression of endothelial adhesion molecules in response to stimuli such as inflammatory molecules, toxic metabolites and hypoxia. The increased expression of adhesion molecules promotes leukocyte interactions with the endothelium, resulting in leukostasis, migration of leukocytes into the tissue and/or endothelial cell apoptosis [1]. Present evidence [2–6] implicates intercellular adhesion molecule (ICAM)-1, which is expressed at relatively high levels on human retinal endothelium [7], as a critical player.

Targeting retinal endothelial ICAM-1 for therapeutic purposes has been entertained [1]. However, ICAM-1 plays an important role in host immune defense and complete blockade would pose the risk of infection. In previously published work [8], we showed that a stimulus-induced increase in ICAM-1, but not its basal level, might be reduced by manipulating gene expression. Small interfering RNA knock-down of the multi-functional transcription factor (TF), nuclear factor κ-light-chain-enhancer of activated B cells (NF-κB)1, in human retinal endothelial cells significantly reduced a tumor necrosis factor (TNF)-α-induced increase in ICAM-1 expression, but did not alter constitutive ICAM-1 expression. Importantly, NF-κB1 knock-down also significantly reduced leukocyte binding to TNF-α-stimulated human retinal endothelial cell monolayers, but did not impact baseline binding.

### Rationale

Specificity is an important consideration for success in planning a treatment approach that involves manipulating ICAM-1 expression. One candidate molecule with a primary function in regulating ICAM-1 expression is ICAM-1-related (ICR), which is a long non-coding (lnc)RNA transcribed from the anti-sense DNA strand overlapping the *ICAM1*-*ICAM4*-*ICAM5* gene cluster on chromosome 19p13.2. This lncRNA was described recently in a publication by Guo et al. [9], who studied a cell line generated from portal vein thrombus of an individual with hepatocellular carcinoma [10]; the investigators showed ICR bound to and stabilized the ICAM-1 transcript, leading to increased ICAM-1 protein expression. Prior to considering ICR blockade as a treatment of retinal vasculopathy, it is essential to understand how disease-relevant stimuli trigger transcription of ICR. To date, however, the ICR promoter has not been characterized. Thus, we undertook a comprehensive in silico analysis of ICR promoter to identify potential TF binding sites (TFBSs), as well as associations between TFBSs and single nucleotide polymorphisms (SNPs). We also verified that human retinal endothelial cells expressed ICR.

## Main text

### Functionally constrained elements in *ICR* gene promoter regions

**Fig. 1:**
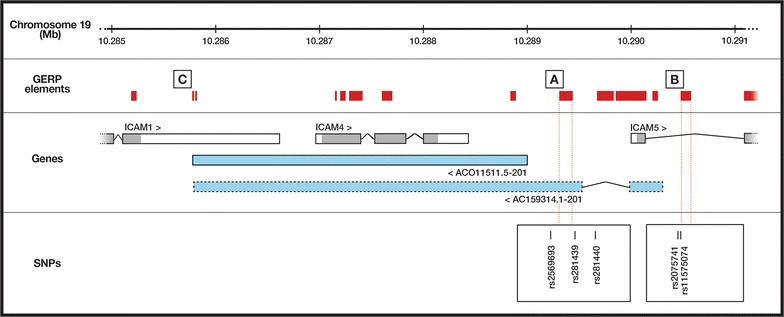
Genomic structure of human ICAM-1-related (ICR) long non-coding RNA. Positions of annotated human ICR gene transcript (AC011511.5-201, solid line: ENST00000589379.1) and predicted alternative transcript (based on annotated mouse sequence: AC159314.1-201, dashed line: ENSMUST00000216917.1), both located on the anti-sense strand, are indicated in relation to positions of *ICAM1* (ENST00000264832.7), *ICAM4* (ENST00000380770.3) and *ICAM5* (ENST00000221980.4) on chromosome 19p13.2 (genome build = GRCh38.p10; grey indicates coding sequence). Genome evolutionary rate profiling (GERP) identifies evolutionarily constrained elements within a region 500 base pairs upstream of the transcription start sites of the human (A: conserved across 25 eutherians) or mouse (B: conserved across 27 eutherians) *ICR* gene sequences, plus two polyadenylation signals at the 3′ end of both *ICR* gene sequences (C: conserved across 26 or 24 eutherians). Phenotype-associated single nucleotide polymorphisms (SNPs) predicted to impact binding of factors that may regulate *ICR* gene transcription are showed in boxes that correspond to GERP-defined elements

Conserved sequences across orthologous promoters may identify TFBSs of functional relevance; conserved sequences signify genomic regions that have resisted evolutionary mutation over time, implying a functional constraint [11]. Sequences for the human *ICR* gene and an orthologous mouse gene have been manually annotated by the HAVANA (Human and Vertebrate Analysis and Annotation) project and lodged with Ensembl [12] (human: AC011511.5 and ENSG00000267607.1; mouse: AC159314.1 and ENSMUSG00000110790). The human gene consists of a single exon that begins in the *ICAM4*-*ICAM5* intergenic region, spans the entire *ICAM4* gene, and overlaps the 3′ untranslated region (UTR) of *ICAM1* (Fig. 1). The mouse gene, which is located on chromosome 9, also spans *Icam4* and overlaps the 3′ UTR of *Icam1*, but has 2 exons: the first exon begins within intron 1 of *Icam5* and continues across exon 1 of *Icam5*, and the second exon begins in the *Icam4*-*Icam5* intergenic region and spans *Icam4* (Fig. 1). Since locations of the transcription start site (TSS) and, by extrapolation, the promoter region, differ between human and mouse genes, we conducted separate analyses to identify regions of constrained DNA sequence across multiple eutherians.

Genomic evolutionary rate profiling (GERP) was performed using the Ensembl genome browser (Ensembl release 89—May 2017) [12] to identify evolutionarily constrained elements across annotated eutherian sequences located within the region 500 base pairs (bp) upstream of the human TSS and the mouse TSS [13]. The human genome build was GRCh38.p10, and the mouse genome build was GRCm38.p5. Genome sources for other eutherians are listed in Additional file 1: Table S1. One GERP-constrained element of 115 bp (located at − 276 to − 390 bp relative to the TSS) in the human *ICR* promoter was common to 25 genomes; the dolphin sequence was excluded, as the match was incomplete across the region. A second GERP-constrained element of 68 bp (located at − 177 to − 244 bp relative to the TSS) in the promoter of the orthologous mouse gene located on chromosome 9 was conserved across 27 genomes; the pika and lesser hedgehog tenrec sequences were similarly excluded due to incomplete matching. The proximity of these conserved, non-coding DNA sequences to the human or mouse TSS indicate a likely role in regulating *ICR* gene expression. Interestingly, the GERP-constrained element in the mouse gene is conserved in the human genome, suggesting a second ICR transcript—with two exons—may exist in the human. Two polyadenylation signals at the 3′ end of the *ICR* gene are also conserved across 26 and 24 eutherian genomes, including human and mouse.

### Transcription factor binding sites within conserved *ICR* gene promoter regions

The GERP-constrained elements—plus 10 bp upstream sequence and 10 bp downstream sequence—within the defined promoter regions of the human *ICR* and orthologous mouse genes were interrogated for predicted TFBSs in the ‘General Core Promoter Elements’ and ‘Vertebrates’ sections of Matrix Library 10.0 (October 2016) using MatInspector [14] in Genomatix Software Suite (http://www.genomatix.de): the ‘Common TFs’ search tool was used; ‘Core similarity’ was set at 0.75; and ‘Matrix similarity’ was set at optimized, to minimize false positives for individual matrices. Searching the human element identified 5 matrix families (containing 15 matrices) common to all 24 eutherian sequences, plus one additional family (containing 2 matrices) in 23 of the 24 sequences. These matrices predicted TFBSs for gene regulators including: HIF1, NRF1, MYC-MAX, BHLHE40, XBP1 and STAF. Searching the mouse element identified one matrix family (containing 8 matrices) in 24 of 25 eutherian sequences, a second family (containing 5 matrices) in 23 of 25 sequences, and a third family (containing 3 matrices) in 19 of 25 sequences. These matrices predicted TFBSs for DNA-binding factors that include: RFX1, AP-1 and BACH2. Matrix families, matrices and predicted binding sites are listed in Table 1, and sequences and locations corresponding to each matrix are provided in Additional file 2: Table S2. The spectrum of TFBSs assigned to the *ICR* gene promoter in our analysis is consistent with transcription in response to a wide range of pathological stimuli that include hypoxia, and metabolic and inflammatory proteins.

**Table 1:** Predicted transcription factor binding sites in evolutionarily conserved elements, as identified by genomic evolutionary rate profiling of the sequence located 500 bp upstream of human ICAM-1-related gene transcription start sites

Human sequence	Matrix family^a^	Matrix^a^	Description
Annotated	V$HIFF	V$ARNT.01	AhR nuclear translocator homodimers
		V$HIF1.02	Hypoxia inducible factor
		V$CLOCK_BMAL1.01	Clock/BMAL1, NPAS2/BMAL1 heterodimers
		V$HRE.02	Hypoxia-response elements
		V$HRE.03	Hypoxia response elements, HIF1a/ARNT heterodimers
	V$HASF	V$HAS.01	HIF-1 ancillary sequence
	V$NRF1	V$NRF1.01	Nuclear respiratory factor
		V$NRF1.02	Nuclear respiratory factor
	V$EBOX	V$MNT.01	MAX binding protein
		V$MYCMAX.02	MYC-MAX binding sites
		V$MYCMAX.03	MYC-MAX binding sites
		V$NMYC.01	N-myc
		V$USF.04	Upstream stimulating factor ½
	V$HESF	V$DEC1.02	Basic helix-loop-helix protein E40 (BHLHE40)
		V$HELT.01	Hey-like transcriptional repressor
	V$CREB	V$XBP1.01	X-box binding protein 1
		V$TAXCREB.02	Tax/CREB complex
	V$STAF	V$STAF.01	Se-Cys tRNA gene transcription activating factor
Predicted (based on mouse)	V$XBBF	V$RFX1.02	X-box binding protein RFX1
		V$RFX2.02	Regulatory factor X, 2
		V$RFX3.01	Regulatory factor X, 3
		V$RFX4.01	Regulatory factor X, 4
		V$RFX4.03	Regulatory factor X, 4
		V$RFX5.02	Regulatory factor X, 5
		V$XBOX.01	Motif bound by RFX proteins
		V$MIF1.01	Myc Intron-binding protein (MIBP)/RFX complex
	V$AP1F	V$AP1.01	Activator protein 1
		V$AP1.02	Activator protein 1
		V$BATF.01	Basic leucine zipper TF
		V$FOSL1.01	Fos-like antigen 1
		V$JUNB.01	Jun-B
	V$AP1R	V$BACH2.02	BTB and CNC homology 1, basic leucine zipper TF 2
		V$MARE_ARE.01	Antioxidant response elements
		V$NFE2.01	NF-E2 p45

^a^Matrix nomenclature is defined by MatInspector [7]. Matrices predict transcription factor binding sites; matrix families group individual matrices that have similar binding properties

### Single nucleotide polymorphisms in proximity to *ICR* gene promoter regions

We sought to identify phenotype-associated SNPs with potential to influence transcription of the human *ICR* gene, searching both the *ICR*-*ICAM5* intergenic region and 100 bp downstream of the *ICR* TSS. We also searched intron 1 of the *ICAM5* gene, since GERP suggested the mouse lncRNA might be represented in the human. Single nucleotide polymorphisms within these regions (plus 25 bp upstream sequence and 25 bp downstream sequence) were extracted from the Single Nucleotide Polymorphism Database of the US National Center for Biotechnology Information [15]. The Genomatix Software Suite SNPInspector was used to search Matrix Library (search settings as described for MatInspector analysis) for promoter elements predicted to be impacted by these SNPs. Results were checked against chromatin immunoprecipitation-sequencing data in ENCODE (Encyclopedia of DNA Elements) [16] to confirm TF binding at SNP locations. This analysis identified 3 SNPs in the defined region between *ICR* and *ICAM5* plus 100 bp downstream of the *ICR* TSS (rs2569693, rs281439 and rs281440), and 2 SNPs in intron 1 of *ICAM5* (rs2075741 and rs11575074) (Fig. 1; Table 2, Additional File 3: Table S3). All these SNPs have been associated with human disease and/or serum levels of the membrane-cleaved form of ICAM-1, which is commonly referred to as soluble (s)ICAM-1.

**Table 2:** Phenotype-associated single nucleotide polymorphisms (SNPs) and predicted impact of minor allele on binding sites for transcription factors within the human ICAM-1-related (ICR) gene promoter

SNP	Alleles	Minor allele		Transcription factors with binding sites in ICR promoter	Impact of minor allele on binding sites	
		Frequency	Associations		Loss	Gain
rs2569693	C/T	0.31	Systemic lupus erythematosus [1] sICAM-1 level [18]	ELF1, POL2, NFKB, MAX, CMYC, E2F6, MXI1, FOXA1, CHD2, HMGN3, ZNF263	PLAGL1, WT1, EGR2, ZKSCAN3, ZBED4, INSM1, MAZR, SALL2, SPZ1	MOK2
rs281439	C/G	0.32	Breast cancer [19]	USF1, USF2, USF3, FOXA1, SIN3AK20, POL2, NFKB, MAX, CMYC, E2F6, MXI1, POL24H8, TCF12, BHLHEAD, HMGN3, IRF1, TBP, YY1, ZNF263	USF	E2F6, GCM1, XCPE
rs281440	A/G	0.30	sICAM-1 level [20]	POL2, TBP, EGR1, SRF, IRF, POL24H8, GATA2, ZNF263	KKLF, ZBTB7, LRRFIP1, E2F4 NM23, MAZR	none
rs2075741	G/C	0.33	Breast cancer [19] Prostate cancer [19] sICAM-1 level [20]	POL2, TAF1, ZNF263	None	DMTE
rs11575074	G/A	0.07	sICAM-1 level [21]	POL2, TAF1, SUZ12, ZNF263	NF1B	MYBL1, MEIS1A_ HOXA9, PTF1

*sICAM-1* soluble intercellular adhesion molecule 1

The rs2569693 SNP has been associated with susceptibility to systemic lupus erythematosus across different human populations [17], and with sICAM-1 levels [18]. Analysis identifies the location of this SNP to coincide with binding sites for numerous TFs, including MYC and SCAN domain family members, and predicts the minor allele will cause loss of sites for MYC family member, MAZR, and SCAN domain family member, ZKSCAN3, plus gain of a site for MOK2. The minor allele at rs281439 has been associated with increased risk of breast cancer and progression of the cancer [19]. Multiple TFs, including USF family members, bind at this position, and the minor allele may code loss of the USF binding site, plus gain of E2F6, GCM1 and XCPE binding sites. The rs281440 minor allele has been linked to reduced levels of sICAM-1 [20]; it is predicted to cause no gains of binding motifs, but loss of several; however, ENCODE identified no TFs that bound this position, and thus conclusions on binding activity are not possible at this time. The minor allele at rs2075741 has been associated with breast and prostate cancer [19], and elevated levels of sICAM-1 [20]. Several TFs are predicted to bind at this site; no TFBS is lost with the minor allele, but a new MTE (designated DMTE in Genomatix Software Suite) binding sequence may be created. Similarly, analysis of rs11575074 minor allele, which also has been linked with elevated sICAM-1 levels [21] reveals no losses in known TFBSs, but sites for MYBL1, PTF1 and MEIS1-HOXA9 may be gained. Our findings suggest at least 4 of 5 SNPs are likely to influence TF binding to the *ICR* gene promoter, and thus alter the level of ICR and ICAM-1 expression.

### Expression of ICR by human retinal endothelial cells

**Fig. 2:**
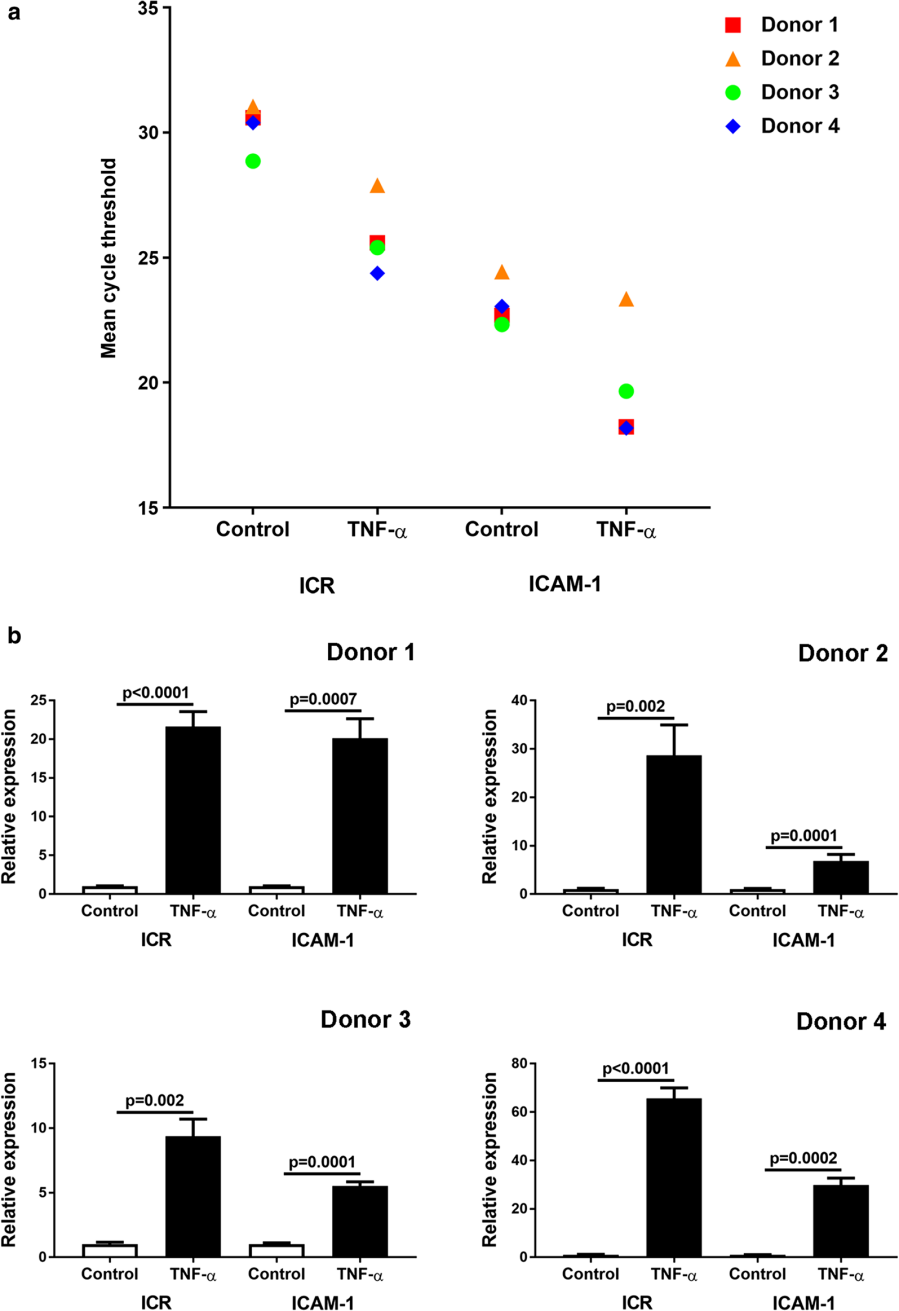
Expression of intercellular adhesion molecule (ICAM)-1-related (ICR) and ICAM-1 by human retinal endothelial cells. Primary endothelial cells were individually isolated from retinae of four different human donors, and exposed to tumor necrosis factor (TNF)-α-supplemented or control medium for 4 h (n = 3 replicate cultures/condition). **a** Graph shows mean cycle threshold values obtained in quantitative real-time polymerase chain reaction for ICR and ICAM-1 cDNA. Results for each human donor are represented by a unique shape, and represent (TNF)-α-supplemented versus control values. **b** Graphs show relative expression of ICR and ICAM-1 transcript by human donor. Bars represent mean relative expression, with error bars showing standard error of the mean. Reference genes were β-actin and glyceraldehyde-3-phosphate dehydrogenase. Data were analyzed by two-tailed Student’s t test

Given that our interest in ICR relates to its potential as a therapeutic target for ICAM-1 in retinal vasculopathy, we wanted to verify the expression and induction of ICR in endothelial cells isolated from human retinae. Tumor necrosis factor-α has been associated with the spectrum of retinal vasculopathies [1], and its signaling pathways involve TFs that recognize the TFBSs identified in our ICR promoter analysis [22, 23]. We generated endothelial cell isolates from four paired human retinae, using the method that we have previously described in detail [1]. Cells were cultured in MCDB-131 medium (Sigma-Aldrich, St. Louis, MO), supplemented with 10% heat-inactivated fetal bovine serum (FBS) (HyClone-GE Healthcare Life Sciences, Logan, UT) and endothelial growth factors (EGM-2 SingleQuots supplement, omitting FBS, hydrocortisone and gentamicin; Clonetics-Lonza, Walkersville, MD) at 37 °C and 5% CO_2_ in air, and used at passage 1 or 2. Confluent cells were treated with fresh medium alone or containing TNF-α (10 ng/mL, R&D Systems, Minneapolis, MN) for 4 h, and lysed with Buffer RLT (Qiagen, Hilden, Germany). Total RNA was extracted using the RNeasy mini kit (Qiagen), and reverse-transcribed using iScript Reverse Transcription Supermix for RT-qPCR (Bio-Rad, Hercules, CA). Quantitative real-time polymerase chain reaction was performed following standard methodology, using iQ SYBRGreen Supermix (Bio-Rad) and on the CFX96 Connect Real-Time PCR Detection System (Bio-Rad) [24]. Relative expression was calculated in the Gene Expression Analysis module of CFX Manager v3.1 (Bio-Rad), which uses the 2^−ΔΔCt^ method [25], normalizing to two stable reference genes. Primer sequences are given in Additional file 4: Table S4. As presented in Fig. 2, we observed that retinal endothelial cell isolates from all four human retinae expressed ICR and ICAM-1. Cellular expression of ICR, as well as ICAM-1 transcript, was significantly increased (p < 0.05: Student *t* test, 2-tailed) following exposure to TNF-α. Both level of ICR expression and degree of induction varied between individual donors.

## Limitations

- Our analysis of the *ICR* gene promoter focused on evolutionarily conserved genomic sequences located within 500 bp upstream of the TSS. While the majority of TFBSs are expected to lie close to the TSS, it is possible that other TFBSs exist outside these regions.
- This investigation was conducted in silico, and therefore it predicts candidate TFBSs, some of which may not be confirmed in biological studies. The computational analysis was comprehensive, but also limited by the capacity of present software tools and database annotations.
- We verified expression and induction of ICR in human retinal endothelial cells, since our interest in this lncRNA relates to the regulation of ICAM-1 protein expression in retinal vasculopathy. Our observations may not apply to all human cell populations.

## Additional files

**Additional file 1: Table S1.** Sources for eutherian genomes used in genome evolutionary rate profiling (GERP) and chromosomal locations of GERP-constrained elements.

**Additional file 2: Table S2.** Predicted transcription factor binding sites within genome evolutionary rate profiling (GERP)-constrained elements.

**Additional file 3: Table S3.** Description of phenotype-nucleotide polymorphisms (SNPs) predicted to influence transcription of the human *ICR* gene.

**Additional file 4: Table S4.** Primer pairs and product sizes for gene transcripts studied in human retinal endothelial cells. References are provided for primers sequences sourced from the literature.

## Abbreviations

Bp: base pairs
FBS: fetal bovine serum
GERP: genomic evolutionary rate profiling
ICAM-1: intercellular adhesion molecule 1
ICR: ICAM-1-related
lncRNA: long non-coding RNA
NF-κB1: nuclear factor κ-light-chain-enhancer of activated B cells
SNP: single nucleotide polymorphism
TF: transcription factor
TFBS: transcription factor binding site
TNF-α: tumor necrosis factor alpha
