# Supplementary material for: ICAM-1-related long non-coding RNA: promoter analysis and expression in human retinal endothelial cells
Source: BMC Res Notes. 2018 May 9;11:285. doi: 10.1186/s13104-018-3384-8 (PMC5944171; doi:10.1186/s13104-018-3384-8)
Supplement: Supplementary file 4 — Additional file 4: Table S4. Primer pairs and product sizes for gene transcripts studied in human retinal endothelial cells. References are provided for primers sequences sourced from the literature. [file 13104_2018_3384_MOESM4_ESM.docx]

Additional file 4: Table S4. Primer pairs and product sizes for gene transcripts studied in human retinal endothelial cells. References are provided for primers sequences sourced from the literature.

| **Gene Transcript** | **Primer Pair** | **Product Size (bp)** |
| --- | --- | --- |
| ICAM1^1^ | Forward: 5’- TAAGCCAAGAGGAAGGAGCA -3’  Reverse: 5’- CATATCATCAAGGGTTGGGG -3’ | 282 |
| ICR | Forward: 5’-CCCAGAAGGTCATAGAAAGTCCGA-3’  Reverse: 5’-TCTAAGCAGCCACAGCCTGAT-3’ | 111 |
| ICAM1+ICR | Forward: 5’- ACAGAGCGAGAGCTTGTCTACA -3’  Reverse: 5’- AAACTGACACCTTTGTTAGCCAC -3’ | 301 |
| ACTB^2^ | Forward: 5’- TCAAGATCATTGCTCCTCCTGAG -3’  Reverse: 5’- ACATCTGCTGGAAGGTGGACA -3’ | 87 |
| GAPDH^3^ | Forward: 5’- AGCTGAACGGGAAGCTCACTGG -3’  Reverse: 5’- GGAGTGGGTGTCGCTGTTGAAGTC -3’ | 209 |

**References**

1. Lu Y, Fukuda K, Nakamura Y, Kimura K, Kumagai N, Nishida T. Inhibitory effect of triptolide on chemokine expression induced by proinflammatory cytokines in human corneal fibroblasts. Invest Ophthalmol Vis Sci*.*2005;46:2346–52.

2. Gutschner T, Baas M, Diederichs S. Noncoding RNA gene silencing through genomic integration of RNA destabilizing elements using zinc finger nucleases. Genome Res. 2011;21:1944-54.

3. Silverman MD, Zamora DO, Pan Y, Texeira PV, Baek SH, Planck SR, Rosenbaum JT. Constitutive and inflammatory mediator-regulated fractalkine expression in human ocular tissues and cultured cells. Invest Ophthalmol Vis Sci*.* 2003;44:1608–15.
